# Rectal Arteriovenous Malformations: A case report of a rare cause of Lower Gastrointestinal Bleeding

**DOI:** 10.1016/j.ijscr.2025.112025

**Published:** 2025-10-06

**Authors:** Amro Mureb, Hamza Daradkeh, Manar Ezzat Abdulmunim Mahmood, Mohammad B.S. Abulqaraya, Ibrahim K.A. Al-Amayreh, Sahar Al-Mustafa

**Affiliations:** a King Hussein Cancer Center, Jordan; b The University of Jordan, Jordan

**Keywords:** Rectal arteriovenous malformations, Rectal AVM, Rectal vascular malformations, Case report

## Abstract

**Introduction and importance:**

Rectal arteriovenous malformation (AVMs) are a rare cause of lower gastrointestinal bleeding. These vascular anomalies, typically present at birth, can progress over time and often lead to significant clinical challenges. The lack of a standardized classification or treatment guidelines further complicate diagnosis and management. This case report aims to contribute to the understanding of rectal AVMs by detailing clinical presentation, diagnosis, and treatment strategies.

**Case presentation:**

We present a case of a 37-year-old male with hematuria and recurrent rectal bleeding, diagnosed through a series of imaging studies, including CT scan and pelvic MRI. Despite undergoing multiple angioembolization procedures, the patient's symptoms persisted, leading to significant anemia and frequent emergency interventions. The decision was made to proceed with a laparoscopic low anterior resection with diverting ileostomy after a thorough discussion of the risk and benefits.

**Clinical discussion:**

This case underscore the complexity associated with diagnosis and managing rectal AVMs. The clinical presentation can vary significantly, and while symptoms often include painless rectal bleeding, many patients experience co-morbid condition due to chronic blood loss. Diagnosis typically involves imaging modalities, with angiography remaining the gold standard. Treatment options vary, and while angioembolization and endoscopic techniques may provide temporary relief, surgical intervention is often necessary for cases resistant to less invasive approaches.

**Conclusion:**

Rectal AVMs represent a rare but significant cause of gastrointestinal bleeding, necessitating a high index of suspicion for diagnosis. This case highlights the challenges in management and the need for further research to establish standardized treatment protocols.

## Highlights

• Rectal arteriovenous malformations (AVMs) is a rare and underreported condition with limited literature and no standardized classification or management guidelines, leading to diagnostic challenges and variability in treatment approaches.
• Rectal bleeding is the most common presenting symptom, with colonoscopy being the primary diagnostic tool.
• Surgical resection, embolization and sclerotherapy are all available treatment modalities with variable success rate.

## 1 Introduction

Rectal arteriovenous malformations (AVMs) are an extremely rare cause of lower gastrointestinal bleeding [1]. AVMs are abnormal blood vessels, usually present at birth and progress over time [1,2]. Clinical presentation is variable and depends on the location and size of the lesion [3]. As of now, rectal AVMs lack a standard classification system and nomenclature [4]. Diagnosis is often done through multiple modalities like MRI and CT scan, with angiography being the gold standard [[4], [5], [6]]. Diagnosis remains a challenge for physicians, often leading to diagnosis delay and misdiagnosis [7]. Rectal AVMs still lack a standardized treatment guideline [8], and follow-up, endoscopic resection, sclerotherapy, angioembolization, medical therapy, and surgery are all possible proposed treatment options with variable success rates [1,4,8,9].

In this article, we aim to report a case of rectal arteriovenous malformation (AVM) encountered in our practice, where we will briefly discuss its presentation, diagnosis, and management. This case report has been reported in line with the SCARE checklist [10].

## 2 Case

**Fig. 1:**
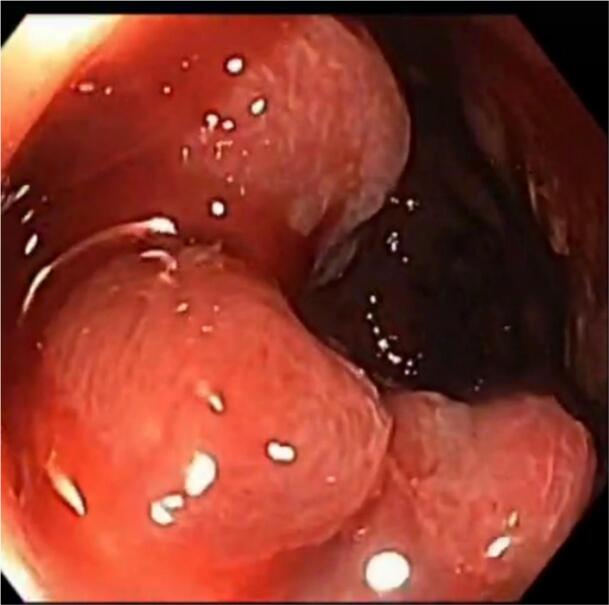
Colonoscopy showed inflamed hyperemic mucosa which is highly vascularized, with diffuse dilated superficial veins in the rectum up to 15 cm from anal verge.

**Fig. 2:**
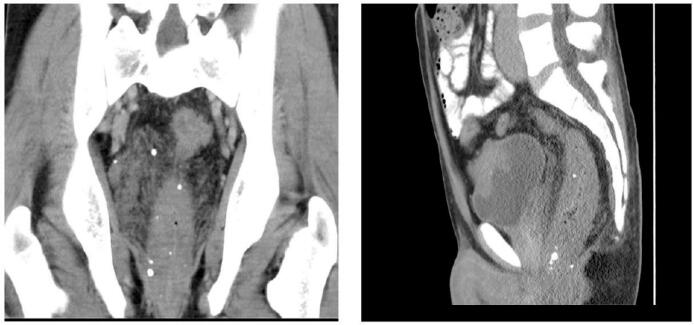
CT scan abdomen and pelvis showed Marked irregular circumferential mural thickening extending from the anorectal wall reaching the distal part of the sigmoid colon, measuring up to 2 cm in thickness and about 15 cm in length, associated with multiple calcifications/phleboliths within the wall and in the mesorectal space along with numerous peri-rectal serpiginous structures.

**Fig. 3:**
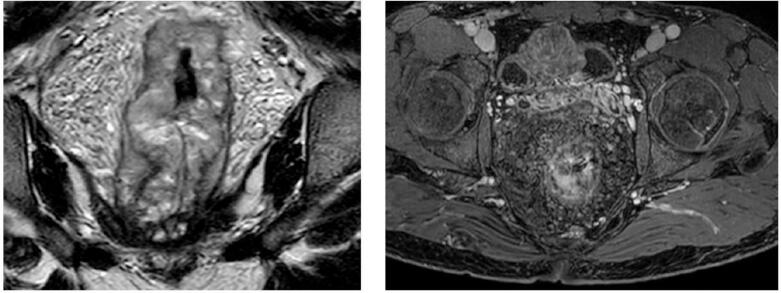
Pelvic MRI scan with marked irregular circumferential mucosal and submucosal thickening involving the anorectal wall, about 2.3 cm from the anal verge, extending into the distal part of the sigmoid colon, measuring about 1.5 cm in thickness and 16 cm in length, associated with numerous serpiginous flow void structures showing enhancement on postcontrast sequences.

**Fig. 4:**
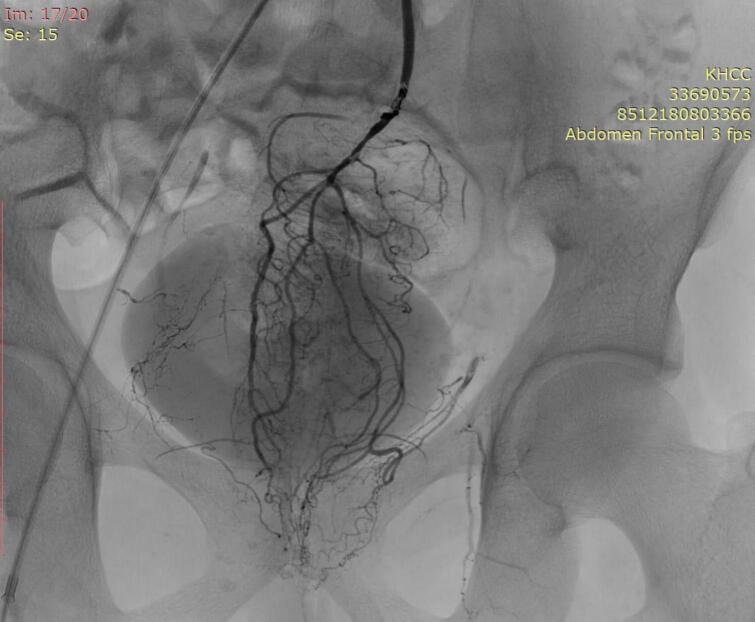
Selective catheterization of the inferior mesenteric artery with contrast injection demonstrates a markedly abnormal vascular pattern in the rectal region. The superior rectal artery is the primary feeder, giving rise to multiple tortuous branches that converge into a compact nidus within the mid-to-lower rectum. Findings are consistent with a rectal arteriovenous malformation, supplied mainly by branches of the superior rectal artery, with early venous drainage into the mesenteric and pelvic venous systems.

**Fig. 5:**
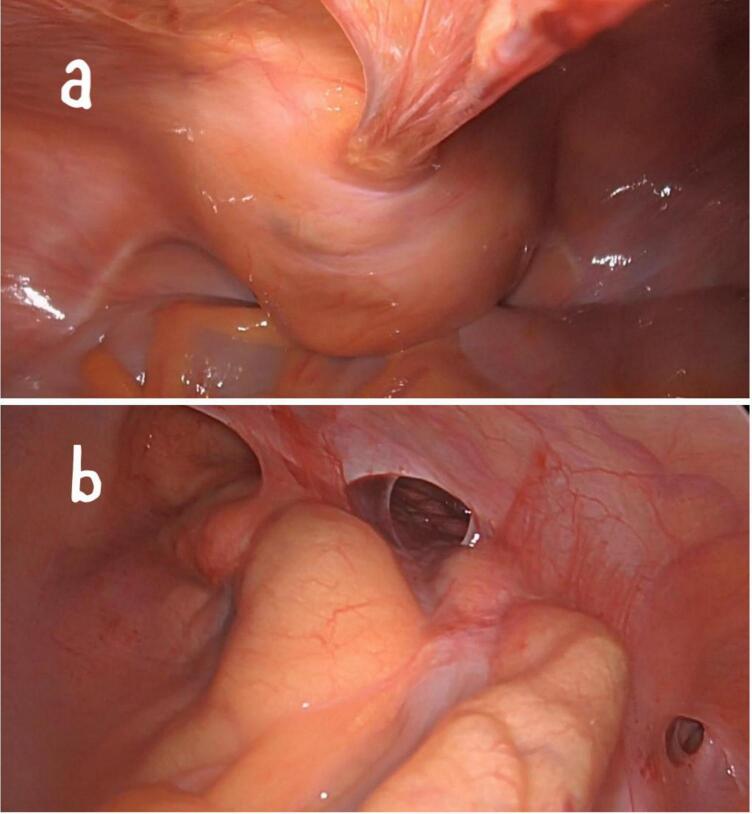
a. Mass at the dome of the urinary bladder. b. Congested hyperemic perirectal tissue.

**Fig. 6:**
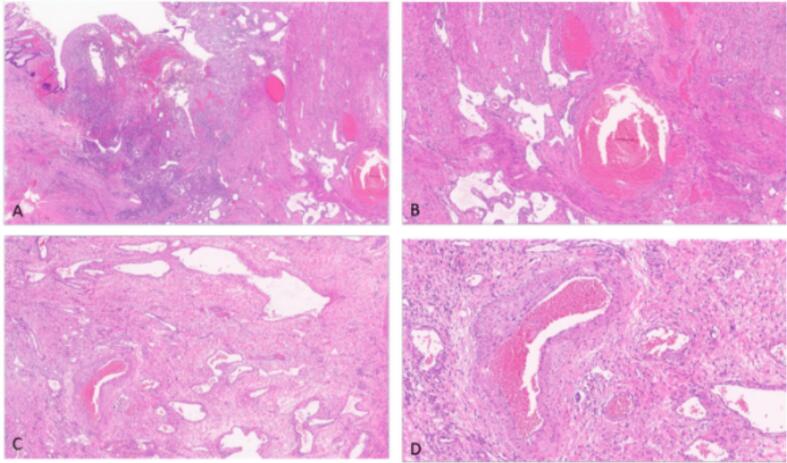
A- 2× magnification H&E section showing mucosal erosion overlying the vascular lesion.B- 4× magnification H&E section showing variably sized blood vessels some of which show large caliber and exhibit thick muscular layer.C- 4× magnification H&E section showing one large muscular vessel with adjacent other thin-walled vessels.D- 10× magnification H&E section showing large blood vessel with thick muscular layer and adjacent other variably-sized vessels.

Our patient is a 37-year-old male patient, previously healthy, who presented to our hospital with hematuria of one year duration and a suspected urinary bladder mass. On further assessment patient reported recurrent fresh rectal bleeding and had a hemoglobin level of 6.0 g/dl. Cystoscopy showed a large mass with hyperemic mucosa arising from the dome of the urinary bladder; however, there was no active bleeding at the time of cystoscopy and bladder biopsy revealed normal urothelial mucosa. Colonoscopy was done showing inflamed hyperemic mucosa with diffuse dilated superficial veins extending up to 15 cm from the anal verge, Fig. 1, no masses were identified, and normal colonic mucosa was seen up to the terminal ileum. Rectal biopsy was not attempted due to the high risk of bleeding. CT scan showed an anterior bladder wall mass with diffuse rectal wall thickening, suggestive of multiple vascular malformations, Fig. 2. Pelvic MRI scan confirmed a 5 cm irregular mass at the anterior bladder wall with severe mucosal thickening and submucosal oedema of the rectosigmoid and upper rectal wall; however, no discrete mass was noted, Fig. 3. In light of the findings, the decision was made to proceed with angioembolization. The patient underwent a total of four embolization procedures, two for the rectal AVM feeders and another two for the bladder mass feeders; however, there was no improvement in rectal bleeding with significant improvement in hematuria symptoms and the urology team decided to keep the patient under follow up for this benign mass. Fig. 4 shows selective catheterization of the inferior mesenteric artery with contrast injection demonstrates a markedly abnormal vascular pattern in the rectal region. Eventually, he developed symptoms that align with Leriche's syndrome, having symptoms of erectile dysfunction, chronic pelvic pain, and gluteal claudication. After a thorough discussion with the patient, the decision was made to proceed with surgery as a last resort, given the persistent, intractable bleeding and frequent emergency room visits for multiple blood transfusions; a total of 13 units. He underwent Laparoscopic low anterior resection with diverting ileostomy, Fig. 5 demonstrates the intraoperative appearance of the bladder mass and upper rectum. One week after surgery, he developed a pelvic collection, which was drained by an interventional radiologist. He was treated conservatively and discharged home uneventfully. Pathology confirmed an arteriovenous malformation, Fig. 6. On immediate post-operative follow-up, the patient was doing well with no new onset of bleeding but with continuous pelvic pain for which he underwent a superior hypogastric block. He also underwent an uneventful stoma reversal. The patient is currently 6 months post-surgery, doing well and free of rectal bleeding.

## 3 Discussion

Rectal arteriovenous malformations are a rare but important cause of lower gastrointestinal bleeding, accounting for only 0.8–2.9 % of cases [1]. These lesions consist of an inflow artery, a collection of abnormal vessels, and an outflow vein that bypasses the normal capillary network [1]. They are characterized by full-thickness involvement of the rectal wall, sometimes extending into mesorectal fat or adjacent structures [8]. AVMs are mostly present at birth and progress over time due to vascular dilation [2]. The clinical presentation varies based on the lesion's size and location [3]. Approximately 10 % of the lesions are asymptomatic and are discovered incidentally [11]. Symptomatic cases usually present with painless fresh rectal bleeding, and may be accompanied by pain, tenesmus, altered bowel habits, or anemia in 60–90 % of cases [1,5]. The pathophysiology of rectal AVMs remains uncertain, with both congenital and acquired mechanisms proposed [8]. Additionally, there is no standard classification system or nomenclature for gastrointestinal AVMs, with authors using different names interchangeably to describe the presence of abnormal vessels [4].

### 3.1 Diagnosis

Diagnosing rectal AVMs is a challenge [7]. Rectal AVMs must be considered in any patient presenting with recurrent or chronic gastrointestinal bleeding [7]. Colonoscopy is regarded as the diagnostic tool for localizing the source of bleeding [4]. Typical findings include submucosal red lesions or mucosal oedema; however, these abnormalities may not always be present [1,8]. Biopsy is generally not recommended, as it carries a risk of severe bleeding in around 25 % of cases; thus, it should be reserved for cases of suspected malignancy only [4]. CT scans have a limited contribution to the diagnosis, with sensitivity ranging from 40 to 78 %, usually showing rectal wall thickening and the presence of phleboliths [6]. CT angiography can be combined with a CT scan to enhance detection [6]. MRI is superior in cases of active bleeding and is useful in determining lesion extension, involvement of adjacent structures, and in follow-up [6,12]. Endorectal ultrasound can detect malformed vessels, but it is difficult to perform in patients with active bleeding [1]. Despite being invasive, angiography is the gold standard for the diagnosis of AVMs [5].

### 3.2 Treatment

There are no standardized treatment guidelines for rectal AVMs [8]. Asymptomatic patients and those with non-progressive lesions may be managed with active surveillance after thorough investigations to exclude other sources of bleeding and malignancy; however, no standardized follow-up protocol currently exists [1,4]. Endoscopic resection or hemostasis is preferred for small, polypoidal lesions, but has a high recurrence rate due to incomplete occlusion of feeding arteries [8,9]. Sclerotherapy is recommended for accessible lower or mid-rectal lesions, punctate bleeding, segmental lesions, or cases with sphincter involvement, though major concerns include incomplete obliteration and agent-related toxicity [4]. Angio-embolization is considered the first-line in several case reports utilizing agents such as coils, gelatin particles, microspheres, polyvinyl alcohol, N-butyl cyanoacrylate, and onyx [5]. It offers the advantages of being both diagnostic and therapeutic, is less invasive with lower morbidity, and can be used as a bridge to surgery or a salvage option [1]. Although angio-embolization has a lower recurrence rate than sclerotherapy, it remains higher compared to surgery [13]. Surgical resection, once regarded as the gold standard, is now recommended as a last resort due to its invasive nature and potential morbidity [1,5]. No standardized surgical procedure exists, with open, laparoscopic, and robotic approaches all suggested [1,4]. The chosen approach should be tailored based on the lesion's location, sphincter involvement, and the patient's overall status [1,9].

## 4 Conclusion

Rectal AVMs are an extremely rare condition, with variable clinical presentations; they lack a standardized classification system and treatment guideline. As of now, angiography is considered the gold standard for diagnosis. Bleeding is the most common presenting symptom and colonoscopy is the primary diagnostic modality used, with upper rectum involvement is the most frequently reported part.

## Consent

Written informed consent was obtained from the patient for publication of this case report and accompanying images. A copy of the written consent is available for review by the Editor-in-Chief of this journal on request.

## Ethical approval

Case reports without clear identification of patients are exempt from IRB approval in our institution.

## Funding

None.

## Author contribution

Amro Mureb: Operator, Study concept, writing the paper, Literature search.

Hamza Daradkeh: Writer, Literature search.

Manar Ezzat Abdulmunim Mahmood: writing the paper, Literature search.

Mohammad B. S. Abulqaraya: writing the paper, Literature search.

Ibrahim K. A. Al-Amayreh: writing the paper, Literature search.

Sahar Al-Mustafa: writing the paper.

## Guarantor

Amro Mureb.

## Research registration number

N/A.

## Conflict of interest statement

None.
